# Racial/Ethnic Differences in Health-Related Quality of Life Among Gay and Bisexual Prostate Cancer Survivors

**DOI:** 10.3389/fonc.2022.833197

**Published:** 2022-04-13

**Authors:** Alex J. Bates, B. R. Simon Rosser, Elizabeth J. Polter, Christopher W. Wheldon, Kristine M. C. Talley, Ryan Haggart, Morgan Wright, Darryl Mitteldorf, William West, Michael W. Ross, Badrinath R. Konety, Nidhi Kohli

**Affiliations:** ^1^Division of Epidemiology and Community Health, School of Public Health, University of Minnesota, Minneapolis, MN, United States; ^2^Department of Social and Behavioral Sciences, College of Public Health, Temple University, Philadelphia, PA, United States; ^3^Adult and Geriatric Health, University of Minnesota School of Nursing, Minneapolis, MN, United States; ^4^Department of Urology, University of Minnesota, Minneapolis, MN, United States; ^5^Malecare Cancer Support, New York, NY, United States; ^6^Department of Writing Studies, University of Minnesota, Minneapolis, MN, United States; ^7^Department of Family Medicine and Community Health, University of Minnesota Medical School, Minneapolis, MN, United States; ^8^Allina Health Cancer Institute, Minneapolis, MN, United States; ^9^Department of Educational Psychology, University of Minnesota, Minneapolis, MN, United States

**Keywords:** prostatic neoplasms, sexual and gender minorities (SGMs), quality of life, ethnic groups/epidemiology, cancer, oncology

## Abstract

**Introduction:**

Prostate cancer treatment has established effects on the health-related quality of life (HRQOL) of patients. While racial/ethnic differences in HRQOL have been explored in heterosexual patients, this is the first study to examine racial/ethnic differences in a cohort of sexual minority prostate cancer survivors.

**Methods:**

We used data from the Restore-1 study, an online cross-sectional survey of sexual and gender minority (SGM) prostate cancer survivors in North America, to explore the association between race/ethnicity and HRQOL. General mental and physical HRQOL was assessed using the Short-Form Health Survey version 2 (SF-12). The frequency and distress of prostate cancer specific symptoms was assessed using the Expanded Prostate Cancer Composite (EPIC) scale. Multivariable linear regression was used to estimate mean differences in HRQOL between sexual minority men of color and their white, non-Hispanic counterparts after adjustment for pertinent demographic and medical characteristics.

**Results:**

Among 190 participants, 23 (12%) self-identified as non-white and/or Hispanic. In unadjusted analysis, sexual minority men of color compared to their white counterparts reported worse HRQOL scores in the EPIC hormonal summary (73.8 vs. 81.8) and hormonal function (70.9 vs 80.5) domains. Clinically important differences between men of color and their white counterparts were seen in the EPIC bowel function (mean difference (MD): -4.5, 95% CI: -9.9, 0.8), hormonal summary (MD: -8.0, 95% CI: -15.6, -0.4), hormonal function (MD: -9.6, 95% CI: -17.6, -1.6), and hormonal bother (MD: -6.7, 95% CI: -14.4, 1.1) domains. After adjustment for covariates, clinically important differences persisted between men of color and white, non-Hispanic men on the hormonal summary (74.4 vs. 81.7), hormonal function (71.3 vs. 80.3), and hormonal bother (77.0 vs. 82.7) domains.

**Conclusions:**

This exploratory study provides the first evidence that sexual minority men of color may have worse HRQOL outcomes compared to white, non-Hispanic sexual minority men following prostate cancer treatment.

## Introduction

Sexual minority men (i.e., those who identify as gay or bisexual) seeking cancer care face greater psychological distress (1), poorer quality of life outcomes (1), increased discrimination (2, 3), and experience significant cancer outcome disparities (2–5) when compared to their heterosexual counterparts. The proportion of adults in the United States (U.S.) who identify as a sexual minority has steadily increased in recent years, with current estimates indicating that 5.6% of U.S. adults identify as such (6). However, while sexual minority communities face disparities in cancer outcomes there is a significant gap in the literature of sexual minority cancer studies, with little known about their unique experiences and needs (2, 7–9).

Prostate cancer is the second most common type of cancer in the U.S. and the most common type of invasive cancer among men (10). In 2021 alone, over 240,000 new cases are estimated to be diagnosed in the U.S (10). The proportion of sexual minority prostate cancer patients is expected to increase from the current level of approximately 2% to 4% in the next decades (11), suggesting that over 100,000 sexual minority men will be living with prostate cancer in the U.S (12). With this increase comes a need for additional research examining the disparities faced within this community to better guide future public health policy and interventions.

Few studies have explored prostate cancer in sexual minority populations (5, 9, 13, 14). Sexual minority men experience more functional (e.g., urinary, bowel, hormonal, sexual) issues following treatment (1, 15–19), as well as lower health-related quality of life outcomes (HRQOL) (1, 13, 15, 16), compared to their heterosexual counterparts. Sexual minority men have worse quality of life outcomes in multiple prostate cancer specific domains, as well as poorer overall mental health when compared to published norms for heterosexual prostate cancer survivors (16). Past research has found older sexual minority men experience more sexual symptoms and greater distress related to these symptoms after treatment (16). Similarly, those who are HIV-positive experience greater urinary, sexual, and bowel symptoms and greater distress related to these symptoms after treatment (20).

Research in the general population has consistently shown significant racial differences in the experiences of prostate cancer in communities of color. Some Asian American subpopulations, particularly those who are foreign-born, are more likely to present with advanced disease (21, 22) and have higher mortality rates (21, 23) compared to their white, non-Hispanic counterparts. Additionally, Asian-American men have worse urinary incontinence in the first year following prostatectomy compared to white, non-Hispanic prostate cancer survivors (24). While American Indian and Native Alaskan men are less likely to be diagnosed with prostate cancer, they also have higher mortality rates compared to white, non-Hispanic men (21, 25).Compared to their white, non-Hispanic counterparts, Black men are more likely to be diagnosed younger (26–29), and have higher mortality rates (10, 29–34). Additionally, Black prostate cancer survivors are more likely to report worse urinary function with slower recovery (35–37), and worse general and mental health (35, 38), compared to white, non-Hispanic survivors. Black and Hispanic men are also more likely to be diagnosed with more advanced cancer (27–29, 39–41), and are less likely to receive definitive treatment (33, 42) when compared to their white, non-Hispanic counterparts. Among prostate cancer patients treated with surgery, Black and Hispanic men are more likely to report worse bowel function (37) and problems with their sexual function (37, 43). However, Black men report better overall sexual function after any type of treatment (37, 43) and better urinary functioning after prostatectomy (43). Whether these findings extend to sexual minority populations has not been studied.

The experiences of sexual minority men of color with prostate cancer have not been previously studied. Globally, there have been only seven other quantitative studies of sexual minority prostate cancer survivors published (1, 15, 17, 18, 44–46) with none being large enough to explore racial/ethnic differences. The experiences of sexual minority prostate cancer survivors can be explained through the lens of minority stress and intersectionality. Minority stress theory suggests that sexual and gender minority (SGM) people experience unique stressors related to their experiences of stigma and discrimination, which results in worse health outcomes (47, 48). Intersectionality theory provides a framework for understanding how multiple social identities intersect to provide individuals with experiences that are distinct from any single identity, reflecting systems of privilege and oppression present in society (49, 50). To fill in the gap in research, we used a cross-sectional survey of sexual minority prostate cancer survivors (16) to quantify racial differences in overall HRQOL and prostate cancer specific HRQOL in sexual minority men who have undergone prostate cancer treatment. Using the theories from above, we hypothesized that since sexual minority men of color face multiple stressors from racism and homophobia (2, 48, 51), they should experience worse HRQOL outcomes after prostate cancer treatment when compared to their white, non-Hispanic counterparts.

## Methods

### Design and Participants

Data were from Restore-1 study which was an online cross-sectional survey conducted in 2015 of 193 gay and bisexual men and one transgender person in the U.S. and Canada who had been treated for prostate cancer (16, 52). Participants were recruited from online advertisements as well as emails sent to Malecare.org, a large North American cancer advocacy organization and support group. Participants completed a brief screening survey and were deemed eligible if they were: (1) a gay, bisexual, or other man who has sex with men, (2) 18 years or older, (3) able to read English, (4) had been treated for prostate cancer before the survey, and (5) living in the U.S. or Canada. Participants who were eligible then went through an informed consent process and, if they consented, were directed to the final survey. Each participant received a $25 gift card as compensation.

The detailed recruitment protocol for this study as well as the cross-validation and de-duplication procedures are described elsewhere (16, 52). In all, 427 surveys were received. Following online survey best practices, surveys and survey response patterns were evaluated for both fraud and duplication, resulting in 233 surveys being deemed invalid or duplicative and one insufficiently complete. These were removed from the final sample. All study procedures were approved by the University of Minnesota institutional review board.

### Measures

Questions from the U.S. Census were used to assess participant demographics such as race, ethnicity, age, and education. One survey item was used to assess the participant’s race (“What is your race?”) with participant’s selecting one or more of the following: American Indian or Alaska Native American, Asian American, Black or African American, Native Hawaiian or other Pacific Islander, White, or Other race. One survey item was used to access ethnicity (“Are you Spanish/Hispanic/Latino?”). Questions related to a participant’s sexual orientation, relationship status, and HIV status were based on prior research conducted by this study’s principal investigators (53, 54). Questions pertaining to prostate-specific antigen (PSA) level at time of diagnosis and Gleason score at time of diagnosis were derived from previous studies conducted on prostate cancer (55, 56). Type of prostate cancer treatment participants had received was assessed by asking participants if they had received any of the following nine treatments: surgery (e.g., radical prostatectomy), external radiation therapy, brachytherapy, cryotherapy, medical castration, surgical castration, diet and/or alternative therapy, and active surveillance. To assess whether participants were taking medications that can have sexual side effects (e.g., loss of sexual interest, erection difficulties) participants were asked if they were taking any of fourteen different medication classes, with the following classes of medications being pertinent for the current study: prostate cancer medications (e.g., Leuprolide) and chemotherapy medications. To measure discrimination encountered by participants during treatment, the Everyday Discrimination Scale (EDS), adapted for medical settings (57), was used. This seven-item scale asks participants the frequency of discrimination they experience during their provider interactions, with higher scores indicating more frequent discrimination (57).

General HRQOL was assessed using the Short-Form Health Survey version 2 (SF-12). The SF-12 contains twelve items answered with Likert scales. It contains two subscales related to mental and physical health. Each domain is normed with a mean score of fifty, with higher scores indicating better health (58). In the general population, the SF-12 has high internal consistency for the physical and mental domains (Cronbach’s α ≥0.72), as well as high test-retest reliability (r ≥ 0.73) (58). Minimal clinically important differences (MCID) between scores (that is, the change in score that would be noticeable to the patient) have previously been reported in general populations of prostate cancer survivors for the SF-36 version of this scale, which has been found to be highly correlated with the SF-12 version (59). These MCID estimates were 6 for the physical function domain and 8.4 for the mental health domain (60).

Prostate-cancer-specific quality of life was assessed using the Expanded Prostate Cancer Index Composite (EPIC) scale. We employed the 50-item version which yields four symptom domains: urinary, bowel, sexual, and hormonal (61). Each domain is further divided into a function and bother subdomain, which assess the frequency of symptoms related to that domain and the distress caused by those symptoms respectively. Each domain and subdomain is scored from 0 to 100, with 100 indicating better health in that particular area. Overall, domain summary scores are the combination of its corresponding functional and bother subdomain scores. In general populations the EPIC scale has high internal consistency (Cronbach’s α ≥ 0.82), test-retest reliability (r ≥ 0.80), and validity with Pearson’s correlation coefficients ranging from 0.29 to 0.77 (61). MCID between scores for the 26-item version of this scale, which is highly correlated with EPIC-50 (62), have previously been estimated for each domain in the general population (63). These MCID estimates were 5–7 for the urinary irritative/obstructive domain, 6–9 for the urinary incontinence domain, 10–12 for the sexual domain, and 4–6 for the hormone and bowel domains (63). MCID estimates for the urinary irritative and urinary incontinence domains were used for the EPIC-50 urinary bother and urinary function domains, respectively (64).

### Analysis

Given the small number of non-white and Hispanic participants, racial and ethnic categories were collapsed into either non-white and/or Hispanic (i.e., men of color) or white, non-Hispanic. This method of combining small numbers of non-white and Hispanic participants into one group is similar to other exploratory studies (65–67). A proxy measure of the current severity of the participant’s cancer was created by summing the two classes of prostate-cancer related medications participants were asked about during the survey, that is chemotherapy medications and prostate cancer medications (e.g., Leuprolide). Participant demographic, medical, and HRQOL characteristics were summarized using means and standard deviations for continuous variables and counts and percentages for categorical variables. Participant characteristics were compared by racial/ethnic group using *t*-tests for continuous variables and chi-square tests or Fisher exact tests, when appropriate, for categorical variables. Descriptive statistics (mean and standard deviation) of each HRQOL measure were calculated for each separate racial/ethnic group (e.g., white, Black/African American, Asian American etc.) to allow for descriptive analysis.

Multivariable linear regression was used to assess the unadjusted mean differences (MD) and adjusted mean differences (AMD) between men of color and white, non-Hispanic men for all EPIC and SF-12 domains. *Post-hoc* power calculations were performed for each measure using each groups sample size, mean, standard deviation, and the corresponding MCID for that measure. Power to detect MCID between scores ranged from 0.22 (EPIC urinary bother domain) to 0.71 (EPIC sexual summary domain) for the EPIC-50, and 0.77 (physical domain) to 0.88 (mental domain) for the SF-12.

Participants were excluded from analysis if they were missing any SF-12 or EPIC domain or subdomain scores (N=2) or if they refused to answer what their race or ethnicity was (N=1).The multivariable models included variables that had a statistically significant (p ≤ 0.05) association with ethnoracial groups. Because cancer severity may lie on the causal pathway between race/ethnicity and HRQOL (16, 27, 29, 31, 39, 68, 69) these measures (type of prostate cancer treatment, Gleason score, and count of systemic prostate cancer therapies) were not included in any models. Mean differences were considered to be statistically significant at *p* < 0.05. All reported *p-*values were two-sided. All analyses were conducted using Stata Statistical Software (StataCorp. 2021. Stata Statistical Software: Release 17. College Station, TX: StataCorp LLC.).

## Results

The final analytic sample consisted of 190 gay and bisexual men who had undergone prostate cancer treatment, had a score for all EPIC and SF-12 domains and responded to both the race and ethnicity survey items. Most participants self-identified as white (N=170, 89.5%) followed by Black/African American (N=9, 4.7%), with those remaining participants self-identifying as Asian American (N=4, 2.1%), other races (“EurAsian” and Hispanic) (N=3, 1.6%), American Indian/Alaska Native American (N=2, 1.1%), or multiracial (American Indian or Alaska Native American and white) (N=2, 1.1%). Participants largely self-identified as non-Hispanic (96.8%) with those remaining identifying as Hispanic (3.2%). Participants who identified as Hispanic were largely Mexican, Mexican American, or Chicano (N=3), followed by Puerto Rican (N=2), and “other” Hispanic ethnicity (N=1). Taken together, 23 (12%) participants self-identified as non-white and/or Hispanic with those remaining 167 (88%) participants being white and non-Hispanic. Participants had a mean age of 63.5 years (SD=8.2). Most participants had at least a bachelor’s degree (77.4%) and over half were married or in a long-term relationship (55.4%). The preponderance of participants self-identified as gay/homosexual (90.5%) with those remaining identifying as bisexual (9.5%).

Participant’s demographic and medical characteristics by race/ethnicity are presented in **Table 1**. Compared to their white, non-Hispanic counterparts, men of color were significantly younger, more likely to be HIV-positive, and more likely to be on one-or-two methods of systemic prostate cancer therapies. No other participant characteristics were significantly different between the two groups.

**Table 1:** Characteristics of Participants by Race and Ethnicity Enrolled in Restore-1: A Survey of Sexual Minority Prostate Cancer Survivors (N=190).

Variables	SGM of color (non-white and/or Hispanic) N=23	White, non-Hispanic SGM N=167	Test of statistical difference (*p*-value)^a^
**Demographics**			
**Age (mean, SD)**	59.8 (8.7)	64.0 (8.0)	0.021
**Education (N(%))**			0.430
Less than Bachelor’s Degree	4 (17.4%)	39 (23.4%)	
Bachelor’s Degree	11 (47.8%)	56 (33.5%)	
Graduate Degree	8 (34.8%)	72 (43.1%)	
**Relationship Status (N(%))**			0.821
Single/Dating/Divorced/Widowed	9 (40.9%)	74 (45.1%)	
Partnered/Married	13 (59.1%)	90 (54.9)	
**Sexuality**			0.999
Gay/Homosexual	21 (91.3%)	151 (90.4%)	
Bisexual or Other	2 (8.7%)	16 (9.6%)	
**Income**			0.579
<$35,000	3 (15.8%)	35 (23.3%)	
$35,000-79,999	9 (47.4%)	52 (34.7%)	
≥$80,000	7 (36.8%)	63 (42.0%)	
**Everyday Discrimination Scale (mean, SD)**	2.7 (4.2)		
**Medical characteristics**			
**HIV Status (N(%))**			0.006
HIV Negative	16 (69.6%)	149 (89.8%)	
HIV Positive	7 (30.4%)	17 (10.2%)	
**Treatment (N(%))**			0.328
Surgery (Only)	9 (40.9%)	89 (54.6%)	
Radiation (only)	4 (18.2%)	31 (19.0%)	
Combined/Systemic	9 (40.9%)	43 (26.4%)	
**Time since diagnosis in years (Mean, SD)**	5.5 (5.0)	5.6 (4.5)	0.919
**PSA at diagnosis (Mean, SD)**	6.2 (6.1)	7.8 (6.6)	0.355
**Gleason score at diagnosis (N(%))**			0.585
≤6	10 (52.6%)	60 (42.9%)	
7	7 (36.8%)	53 (37.9%)	
8-10	2 (10.5%)	27 (19.3%)	
**Count of methods of systemic prostate cancer therapies (N(%))**			<0.001
No medications taken for prostate cancer	16 (69.6%)	142 (85.0%)	
One method of systemic prostate cancer therapy (either chemotherapy or prostate cancer medications such as Leuprolide)	5 (21.7%)	25 (15.0%)	
Two methods of systemic prostate cancer therapies (both chemotherapy and prostate cancer medications such as Leuprolide)	2 (8.7%)	0	

a Participant characteristics were compared using t-tests for continuous variables and chi-square tests or Fisher exact tests, when appropriate, for categorical variables.

Descriptive statistics of the HRQOL measures (EPIC and SF-12) by each separate racial and ethnic group are reported in **Table 2**. While white, non-Hispanic men had higher or similar mean scores on all domains compared to all other ethnoracial groups, Latino men had the highest mean scores on all EPIC urinary and bowel domains as well as the SF-12 mental function domain. All other ethnoracial groups consistently had similar or lower mean scores compared to white, non-Hispanic men and Latino men.

**Table 2:** Descriptive Statistics of Health-Related Quality of Life (HRQOL) outcomes for Sexual Minority Prostate Cancer Survivors by Racial and Ethnic Group (N=189).

	White, non-Hispanic (N=167)	Black/African American (N=8)	Latino (N=6)	Asian American (N=4)	American Indian/Alaska Native American (N=2)	Multiple races^a^ (N=2)
**EPIC-50**^**b**^ **(Mean, SD)**						
**Urinary summary**	77.7 (16.9)	67.3 (33.1)	92.0 (9.7)	65.1 (29.4)	77.1 (26.5)	54.9 (23.6)
Function	82.0 (17.8)	72.1 (36.1)	93.3 (13.4)	72.5 (26.3)	65.0 (42.4)	61.7 (28.4)
Bother	74.6 (19.8)	63.8 (31.8)	91.1 (10.3)	59.8 (32.9)	85.7 (15.2)	50.0 (20.2)
**Sexual summary**	45.8 (21.8)	39.0 (23.3)	38.4 (23.4)	41.6 (9.7)	28.4 (15.8)	40.7 (15.0)
Function	41.2 (22.9)	36.8 (21.7)	33.1 (21.2)	35.0 (25.7)	19.4 (7.9)	38.0 (15.7)
Bother	55.7 (24.1)	43.8 (28.5)	50.0(31.9)	56.3 (29.8)	46.9 (30.9)	46.9 (13.3)
**Bowel summary**	87.0 (12.8)	83.0 (16.8)	95.2 (3.9)	73.2 (21.1)	78.6 (25.3)	80.4 (15.2)
Function	89.5 (11.4)	85.3 (15.7)	92.9 (6.0)	74.1 (22.1)	69.6 (32.8)	89.3 (15.2)
Bother	84.5 (16.2)	80.8 (18.5)	97.6 (2.9)	72.3 (22.7)	87.5 (17.7)	71.4 (15.2)
**Hormonal summary**	81.8 (16.7)	74.7 (27.6)	74.6 (17.5)	63.1 (18.6)	71.6 (30.5)	80.7 (1.6)
Function	80.5 (17.5)	71.9 (30.3)	75.0 (14.8)	61.3 (19.3)	60.0 (42.4)	75.0 (7.1)
Bother	82.9 (16.9)	77.1 (27.3)	74.3 (21.3)	64.6 (21.1)	81.3 (20.6)	85.4 (8.8)
**SF-12** ^**c**^ **(Mean, SD)**						
Physical function	52.5 (8.6)	50.5 (14.6)	56.1 (5.9)	55.7 (6.9)	42.5 (10.4)	50.0 (1.5)
Mental function	46.1 (11.4)	48.5 (14.5)	44.5 (6.7)	36.4 (16.4)	50.8 (9.3)	50.8 (9.3)

a Multiple races=American Indian/Alaska Native American and white.

b EPIC-50=Expanded Prostate Cancer Index Composite (scores ranging from 0-100, higher scores indicate better function/less bother).

c SF-12=Short-form health survey (normed with mean 50, with higher scores indicating better HRQOL).

d “Other” races were excluded as this only applied for N=1 participant who identified as “EurAsian”.

HRQOL measures are reported in **Tables 3** and **4**. In unadjusted analysis, men of color were significantly more likely to report worse mean scores on the EPIC hormonal summary (73.8 vs. 81.8, p=0.038) and hormonal function (70.9 vs. 80.5, p*=*0.019) domains, when compared to white, non-Hispanic men. Across all measures, men of color consistently had worse scores on the EPIC and SF-12, though no other measures reached statistical significance. However, mean differences on the EPIC bowel function (MD: -4.5, 95% CI: -9.9, 0.8), hormonal summary (MD: -8.0, 95% CI: -15.6, -0.4), hormonal function (MD: -9.6, 95% CI: -17.6, -1.6), and hormonal bother (MD: -6.7, 95% CI: -14.4, 1.1) domains all reached MCID thresholds. After adjustment for covariates, men of color were still statistically more likely to report worse scores on the EPIC hormonal function domain (AMD: -9.0, 95% CI: -17.3, -0.8) when compared to white, non-Hispanic men. Additionally, men of color reported clinically worse scores on the hormonal summary (74.4 vs. 81.7), hormonal function (71.3 vs. 80.3), and hormonal bother (77.0 vs. 82.7) domains compared to their white, non-Hispanic counterparts. There were no other clinically-or-statistically significant differences between men of color and white, non-Hispanic men on any other EPIC or SF-12 domains.

**Table 3:** Unadjusted Health-Related Quality of Life (HRQOL) outcomes for Sexual Minority Prostate Cancer Survivors by Racial and Ethnic Group (N=190).

	Men of color	White, non-Hispanic men	Mean difference [95% CI]
**EPIC-50** ^a^ **(Mean predicted value [95% CI])**			
**Urinary summary**	74.3 [66.8, 81.8]	77.7 [74.9, 80.5]	-3.4 [-11.4, 4.7]
Function	76.8 [68.9, 84.8]	82.0 [79.0, 84.9]	-5.1 [-13.6, 3.3]
Bother	72.5 [63.9, 81.1]	74.6 [71.4, 77.8]	-2.1 [-11.3, 7.1]
**Sexual summary**	40.8 [31.8, 49.7]	45.8 [42.5, 49.2]	-5.1 [-14.6, 4.5]
Function	36.3 [26.9, 45.7]	41.2 [37.7, 44.7]	-4.9 [-14.9, 5.1]
Bother	50.5 [40.4, 60.7]	55.7 [51.9, 59.4]	-5.1 [-15.9, 5.7]
**Bowel summary**	84.6 [79.2, 90.1]	87.0 [85.0, 89.0]	-2.4 [-8.2, 3.4]
Function	84.9 [79.9, 89.9]	89.5 [87.6, 91.3]	-4.5 [-9.9, 0.8]
Bother	84.3 [77.6, 91.1]	84.5 [82.0, 87.0]	-0.2 [-7.4, 7.0]
**Hormonal summary**	73.8 [66.7, 80.9]	81.8 [79.2, 84.5]	-8.0 [-15.6, -0.4]*
Function	70.9 [63.4, 78.4]	80.5 [77.7, 83.2]	-9.6 [-17.6, -1.6]*
Bother	76.3 [69.0, 83.5]	82.9 [80.2, 85.6]	-6.7 [-14.4, 1.1]
**SF-12** ^**b**^ **(Mean predicted value, 95% CI)**			
Physical function	52.3 [48.6, 55.9]	52.5 [51.2, 53.9]	-0.3 [-4.1, 3.6]
Mental function	45.9 [41.2, 50.6]	46.1 [44.3, 47.8]	-0.2 [-5.2, 4.9]

^*^p<0.05.

a EPIC-50=Expanded Prostate Cancer Index Composite (scores ranging from 0-100, higher scores indicate better function/less bother).

b SF-12=Short-form health survey (normed with mean 50, with higher scores indicating better HRQOL).

**Table 4:** Adjusted Health-Related Quality of Life (HRQOL) outcomes for Sexual Minority Prostate Cancer Survivors by Racial and Ethnic Group (N=189).

	Men of color	White, non-Hispanic men	Adjusted Mean difference [95% CI]^a^
**EPIC-50** ^**b**^ **(Mean predicted value [95% CI])**			
**Urinary summary**	75.7 [68.1, 83.3]	77.4 [74.7, 80.2]	-1.7 [-9.9, 6.4]
Function	78.2 [70.1, 86.4]	81.7 [78.7, 84.6]	-3.4 [-12.1, 5.3]
Bother	73.9 [65.2, 82.5]	74.4 [71.2, 77.5]	-0.5 [-9.8, 8.8]
**Sexual summary**	40.6 [31.6, 49.5]	45.8 [42.6, 49.1]	-5.2 [-14.8, 4.3]
Function	35.3 [26.0, 44.6]	41.3 [37.9, 44.7]	-6.0 [-16.0, 4.0]
Bother	51.9 [41.7, 62.2]	55.5 [51.7, 59.2]	-3.5 [-14.5, 7.5]
**Bowel summary**	85.8 [80.2, 91.3]	86.8 [84.8, 88.9]	-1.1 [-7.0, 4.9]
Function	86.3 [81.2, 91.4]	89.3 [87.5, 91.2]	-3.0 [-8.5, 2.5]
Bother	85.2 [78.3, 92.1]	84.3 [81.8, 86.9]	0.8 [-6.6, 8.3]
**Hormonal summary**	74.4 [67.1, 81.7]	81.7 [79.0, 84.3]	-7.3 [-15.1, 0.6]
Function	71.3 [63.6, 79.0]	80.3 [77.5, 83.1]	-9.0 [-17.3, -0.8]*
Bother	77.0 [69.6, 84.5]	82.7 [80.0, 85.4]	-5.7 [-13.7, 2.3]
**SF-12** ^c^ **(Mean predicted value, 95% CI)**			
Physical function	51.7 [48.2, 55.2]	52.5 [51.3, 53.8]	-0.8 [-4.6, 3.0]
Mental function	47.5 [42.7, 52.2]	45.9 [44.1, 47.6]	1.6 [-3.5, 6.7]

a Adjusted for: age and HIV-status.

b EPIC-50=Expanded Prostate Cancer Index Composite (scores ranging from 0-100, higher scores indicate better function/less bother).

c SF-12=Short-form health survey (normed with mean 50, with higher scores indicating better HRQOL).

*p < 0.05.

## Discussion

In this cross-sectional study of 190 sexual minority prostate cancer survivors, men of color reported worse HRQOL in bowel function and all hormonal domains when compared to their white, non-Hispanic counterparts. However, the clinically significant association found in the bowel function domain, as well as the statistically significant association found in the hormonal summary domain, were explained by differences in HIV status. This finding is consistent with HIV disproportionally impacting sexual minority men of color (70) and compounding the impact of cancer treatment on HRQOL (20).However, after controlling for HIV status, clinically significant disparities persisted on all EPIC hormonal domain measures and statistically significant disparities persisted on the EPIC hormonal function domain.

These findings are consistent with the broader literature on intersectional stress in SGM populations. SGM people of color are exposed to greater stressors when compared to white SGMs (71–73) and report lower levels of HRQOL (74–76). Since significant differences remained after controlling for covariates, the association between worse HRQOL and race/ethnicity is robust in nature. The lack of widespread HRQOL disparities in this study may be explained by the concept of resiliency which refers to a person’s quality of being able to overcome stressful and traumatic situations (2, 48, 77). Past research has suggested that SGM people of color develop unique resiliency skills to cope with racism they face in their daily lives (48, 51, 78, 79). Additionally, older SGM individuals may share common experiences (e.g., living through the height of the HIV/AIDS pandemic) that provided opportunities for this entire generational cohort to build resilience (80). Therefore, differences between racial/ethnic groups might be masked by this commonality.

Future research into sexual minority prostate cancer survivors should aim to better elucidate the unique experiences of people of color by over-sampling racial and ethnic minorities. Specifically, more studies are needed with enough Black sexual minority prostate cancer patients to find whether disparities seen in heterosexual men extends to sexual minority populations. Such studies should also explore the role of resiliency in their data.

### Study Limitations

This study has several limitations that should be taken into consideration. First, only 23 (12%) participants identified themselves as men of color and the total sample size of the study was small. This resulted in an underpowered study and imprecise estimates with wide confidence intervals and an inability to investigate heterogeneity across racial/ethnic groups. We caution that the absence of a significance difference on any measure should not be misinterpreted as a finding of absence. It could simply denote a lack of power. Second, while a strong cross validation and deduplication protocol was used to detect invalid surveys, it is still possible that erroneous surveys were included in this online study. Third, combining all men of color into one group implies homogeneity and may obscure differences. Fourth, the political landscape for sexual minority groups has changed considerably since this data was collected in 2015. These changes could have a meaningful impact on the HRQOL of sexual minority prostate cancer survivors. Fifth, this sample was highly educated, gay, cisgender (i.e., identifying as the gender that was assigned at birth), and living in the U.S. or Canada. We caution these results may not generalize to those less educated, non-cisgender, and residents of other countries.

### Conclusion

This current exploratory study is the first to explore HRQOL racial differences in a population of sexual minority prostate cancer survivors. After adjustment for covariates sexual minority men of color reported worse HRQOL scores on all measures when compared to white, non-Hispanic men. Future research with more granular data examining racial/ethnic differences within this sexual minority community is warranted.

## Data Availability Statement

The datasets presented in this study can be found in online repositories. The names of the repository/repositories and accession number(s) can be found below: https://www.openicpsr.org/openicpsr/project/137241/version/V1/view.

## Ethics Statement

The studies involving human participants were reviewed and approved by Institutional Review Board at the University of Minnesota. The patients/participants provided their written informed consent to participate in this study.

## Author Contributions

CRediT Taxonomy. Conceptualization, DM, BR, and AB. Methodology, BR, NK, EP, MW, and BK. Validation, NK and EP. Formal analysis, AB and BR. Investigation, BR, EP, MW, RH, and NK. Data curation, MW and NK. Writing – original draft, AB and BR. Writing – review and editing, all authors. Supervision, BR and NK. Project administration, BR, BK, MW, and NK. Funding acquisition, BR, KT, CW, WW, DM, MR, and BR. All authors contributed to the article and approved the submitted version.

## Funding

The Restore study was funded by the Division of Cancer Prevention, National Cancer Institute (NCI) [grant number 1 R21 CA182041]: “Understanding the Effects of Prostate Cancer on Gay and Bisexual Men” (CA182041; PI: BR) and the American Cancer Society Institutional Research Grant.

## Author Disclaimer

The content is solely the responsibility of the authors and does not necessarily represent the official views of the National Institutes of Health. This analysis was conducted by the first author as an independent learning experience in partial completion of the requirements for his Masters in Public Health.

## Conflict of Interest

The authors declare that the research was conducted in the absence of any commercial or financial relationships that could be construed as a potential conflict of interest.

## Publisher’s Note

All claims expressed in this article are solely those of the authors and do not necessarily represent those of their affiliated organizations, or those of the publisher, the editors and the reviewers. Any product that may be evaluated in this article, or claim that may be made by its manufacturer, is not guaranteed or endorsed by the publisher.
